# Integrated targeted and untargeted analysis of polar peptides in foods using hydrophilic interaction liquid chromatography–data-independent acquisition–mass spectrometry

**DOI:** 10.1007/s00216-026-06322-7

**Published:** 2026-01-28

**Authors:** Boudewijn Hollebrands, Germaine Thong, Hans-Gerd Janssen

**Affiliations:** 1 Unilever Foods Innovation Centre-Hive, Bronland 14, 6708 WH, Wageningen, the Netherlands; 2 Wageningen University & Research, Laboratory of Organic Chemistry, Wageningen, the Netherlands

**Keywords:** Food peptides, HILIC-DIA**-**MS, Polar bioactive compounds, Retrospective analysis, Untargeted profiling

## Abstract

A hydrophilic interaction liquid chromatography–data-independent acquisition mass spectrometry (HILIC–DIA-MS) workflow was developed for simultaneous targeted semi-quantification of polar peptides and untargeted profiling of both peptides and other polar compounds in complex food matrices. The method uses a zwitterionic HILIC column optimized for separation of short polar peptides that are challenging to retain on reversed-phase columns. Many of these peptides contain charged amino acids and contribute to basic taste modalities such as umami and saltiness. Both targeted peptide analysis and comprehensive untargeted profiling were achieved by applying DIA-MS detection. This data acquisition mode was shown to be reproducible and sensitive while enabling retrospective data processing. High-resolution MS1 scans (60.000 FWHM), combined with fast MS2 scans and DIA mass windows of 15 *m/z* yielded highly repeatable and selective LC–MS profiles, allowing differentiation of structural isomers (e.g., alpha-glutamyl (umami) and gamma-glutamyl (kokumi)). The method was validated using taste-relevant dipeptides, demonstrating low detection limits (0.1–0.9 µM), good intra-day and inter-day precision, and high recovery (96%) in commercial soy sauce and yeast extract matrices. The workflow was further applied to the relative quantification of peptides and the untargeted profiling of characteristic molecular features in cheese, ham, and extracts from dried food ingredients. The integration of targeted and untargeted analyses demonstrates the suitability of HILIC–DIA-MS for comprehensive characterization of polar compounds in food systems.

**Graphical Abstract:**

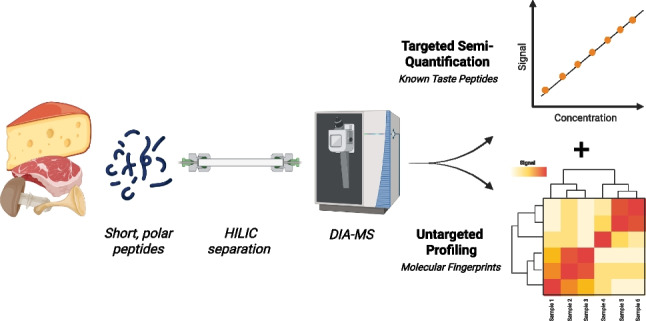

**Supplementary Information:**

The online version contains supplementary material available at 10.1007/s00216-026-06322-7.

## Introduction

In the development of tasty foods, comprehensive molecular profiling is essential to better understand how composition affects taste [1–5]. Within this context, the quantification of taste-relevant peptides is particularly important. In foods, peptides can contribute to a wide range of taste sensations, including bitterness, sourness, sweetness, umami, and saltiness [6]. These taste effects are closely linked to the peptide’s properties, which are primarily determined by its amino acid composition and sequence. However, the comprehensive analysis of these taste-active peptides remains challenging, especially for those that are polar, short, or present at very low levels.

Peptides responsible for key taste attributes such as sourness, salty impression, and umami taste are typically short and polar, containing charged amino acids, such as glutamic acid (E) and aspartic acid (D). For example, the dipeptide Glu-Glu (glutamyl-glutamate), known for its umami taste in fermented foods [7], is highly polar. Due to their strong hydrophilic nature, such short polar peptides are difficult to analyze using conventional reversed-phase liquid chromatography (RPLC) methods. Other taste-active polar compounds such as organic acids, nucleotides, and amino acids have traditionally been analyzed using ion-pairing LC [8]. More recently, hydrophilic interaction liquid chromatography (HILIC) has also been applied [9–11]. However, the application of these techniques to complex mixtures of polar peptides, especially for comprehensive profiling, remains underexplored. This is among others due to the difficulty in optimizing HILIC separation conditions, which involves balancing a multitude of interdependent parameters such as mobile phase composition, buffer strength, and pH.

Most existing studies focusing on polar peptides rely on targeted approaches for quantifying known taste-active peptide sequences [12]. For example, HILIC-triple quadrupole (QqQ) MS has recently been used for targeted quantification of various umami-tasting peptides and γ-glutamyl peptides in a wide variety of different cheeses [13]. Although these targeted methods are robust and sensitive, the use of QqQ MS detection restricts their application to only pre-selected peptides and prevents retrospective untargeted peptide profiling. As a result, potentially important taste-active polar peptides and other markers of food quality and taste may remain undetected.

Recently, He (2025) [7] and Chen (2024) [14] applied untargeted Data-Dependent Acquisition (DDA) MS combined with reversed-phase chromatography separation to profile umami peptides and other taste compounds in soy sauces. However, since taste-active peptides are often present at low levels and can exist as multiple isomeric or isobaric forms (e.g., Leu-Gly vs. Gly-Leu), DDA methods that use the MS1 signal intensity for further MS/MS experiments and quantification are prone to interferences and have a higher risk of missing minor constituents [15].

To overcome these limitations, Data-Independent Acquisition (DIA)-MS strategies have been developed, offering broad and unbiased detection [16, 17]. DIA-MS methods have shown great promise in the analysis of bitter food peptides [18]. Unlike targeted approaches, DIA-MS enables comprehensive spectral data acquisition across a wide *m/z* range. Nevertheless, successful implementation requires careful optimization of the chromatographic conditions and the DIA-MS method parameters, especially if isobaric peptides need to be distinguished. This will often be the case in the analysis of small peptides.

To overcome the limitations of targeted quantification and the incomplete coverage of DDA-based untargeted methods, we here present a validated high-throughput workflow for profiling short polar peptides in food extracts using HILIC-DIA-MS. Conditions for HILIC separation on a zwitterionic stationary phase and DIA acquisition parameters were systematically optimized to enable both targeted and untargeted peptide analysis in selected food matrices. The resulting method was validated and applied to a range of food extracts, allowing not only quantification of known key taste-relevant peptides but also untargeted profiling of other polar constituents that serve as important markers of food quality and taste.

## Material and methods

### Chemicals

Ammonium formate (≥ 99%, LC–MS grade) was purchased from Sigma-Aldrich (St. Louis, MO, USA). Trifluoroacetic acid (99%, LC–MS grade) was purchased from Fisher Chemicals (Landsmeer, the Netherlands). Acetonitrile, methanol absolute, and formic acid of ULC/MS grade were purchased from Biosolve BV (Valkenswaard, the Netherlands). Ultrapure water (resistivity ≥ 18.2 MΩ·cm) was prepared by a Q-POD ultrapure water remote dispenser system from Millipore (Burlington, MA, USA). Dipeptide standards gamma-EE (γEE), gamma-ET (γET), gamma-EF (γEF), gamma-EI (γEI), EE, EK, EV, EL, EA, ED, DA, VF and pyro-EP (pEP) were purchased from Sigma-Aldrich with reported purities ranging from 96 to 100%. The peptides VY, VP, and HL were purchased from Bachem (Bubendorf, Switzerland), and gamma-EM (γEM) and AY were purchased from Pepscan (Lelystad, the Netherlands).

### Standard preparation

Individual peptide standards were prepared by accurately weighing 5 mg of each compound and dissolving it in 10 mL of 50:50 (v/v) methanol:ultrapure water. From each resulting solution, 1 mL was pooled to create a dipeptide stock solution. This stock solution was further diluted with methanol:ultrapure water (50:50, v/v) to prepare calibration curves for semi-quantitative analysis.

### Sample preparation of food samples

A selection of commercial food samples (i.e., Roquefort cheese, Parmigiano Reggiano, Prosciutto crudo ham, serano gran reserva dry-cured ham, and soy sauce) was purchased from local supermarkets. Commercial yeast extract was obtained from a local market. A series of dried food ingredients (e.g., antler mushroom, bonito flakes, seaweed, shiitake mushroom, monkey head mushroom, and dried squid) was purchased from a local Asian supermarket. A detailed list of products and product attributes can be found in supplementary Table S1. All sample extracts were prepared with the procedures listed below, and extracts were stored at −80 °C until analysis.

#### Preparation of cheese extracts

Peptide extraction from Roquefort cheese (Roq) and Parmigiano Reggiano cheese (Par) was performed using a protocol adapted from Toelstede and Hoffman [19]. Extraction was performed in triplicate. For each extract, 5 g of cheese was cut into small pieces, put in a falcon tube (50 mL) with ultrapure water (30 mL), homogenized for 5 min using an Ultra-Turrax, then centrifuged at 4.000 × g for 20 min at 4 °C using a Sigma 3-18KS centrifuge (Sigma, Zwijndrecht, the Netherlands). The upper solid fat layer as well as the protein pellet was removed to leave the liquid layer. Soluble casein was precipitated upon adjusting the pH from 5.3 to 4.6 with formic acid. After a centrifugation step (17.000 × g, 20 min at 4 °C), the supernatant was filtered through a syringe filter (0.2 µm, PTFE, Sartorius, Göttingen, Germany) and used for analysis.

#### Preparation of ham extracts

Adapted from Ju et al. [20], peptides from serrano ham (Ser) and prosciutto ham (Pros) were extracted in triplicate. For each extract, 5 g of ham was added into 25 mL of 0.1% trifluoracetic acid and vortexed for 30 s. After allowing to settle for 1 h at 4 °C, the samples were centrifuged for 20 min at 4.000 × g and 4 °C, using a Sigma 3-18KS centrifuge (Sigma). The supernatant (1 mL extract) was transferred into an empty tube, centrifuged (17.000 × g, 20 min at 4 °C), and finally filtered through a syringe filter (0.2 µm, PTFE, Sartorius).

#### Preparation of yeast extract

A solution of 1% yeast extract was prepared; 100 mg was accurately weighed and added to 10 mL of 50/50 (v/v) methanol:ultrapure water. After mixing, the solution was centrifuged (17.000 × g, 3 min at 4 °C), and the supernatant was used for analysis.

#### Preparation of soy sauce

One gram of soy sauce was added to 10 mL of 50/50 (v/v) methanol:ultrapure water and centrifuged (17.000 × g, 3 min at 4 °C). The supernatant was used for analysis.

#### Preparation of dried food extracts

For each dried food ingredient, 10 g was accurately weighed and added to 350 mL of water. After hydration for 5 h, the soaked materials were filtered through a sieve, leaving clear aqueous extracts. All water extracts were centrifuged for 5 min at 4.000 × g and 4 °C, and the supernatant was used for analysis.

### Instrumentation and conditions

All analyses were performed on a Vanquish UHPLC chromatography system coupled to an Exploris 480 Orbitrap mass spectrometer (Thermo Fisher Scientific, Waltham, MA, USA). The system was equipped with an Atlantis Premier BEH Z-HILIC 1.7 µm, 2.1 × 100 mm column (Waters, Etten-Leur, the Netherlands), along with a Vanguard FIT cartridge pre-column of 2.1 × 5 mm. Optimization of the HILIC conditions was performed through the analysis of a set of dipeptides standards (i.e., VP, VY, HL, γEM, and AY), a soy sauce extract, and a yeast extract. Injection volumes ranging from 0.5 to 8 µL were tested, corresponding to 0.25 to 4% of the total column volume. Considering the pH stability range of the Z-HILIC column (pH 2–10) [21], mobile phase pH values between 3 and 9 were evaluated (i.e., pH 3, 3.8, 5, 7 and 9) to span both acidic and basic conditions. Since pH 3.8 matches the pKa of formic acid, it was included as a separate test condition. Flow rates of 0.3, 0.4, 0.5, and 0.6 mL/min were systematically evaluated. Buffer concentrations of 5, 10, 20, and 40 mM were investigated, and column temperatures of 30, 40, 50, and 60 °C were assessed to determine their influence on performance.

The optimized HILIC conditions included an injection volume of 2.0 µL, a flow rate of 0.5 mL/min, and a column temperature of 50 °C. Solvent A consisted of 10 mM ammonium formate in ultrapure water. Solvent B was 10 mM ammonium formate in 95% ACN–ultrapure water. The pH of both solvents was set to pH 3.8 with formic acid prior to the addition of acetonitrile (solvent preparation procedure can be found in the supplementary materials). A gradient separation was applied starting at 95% B, decreasing to 40% B in 7.5 min, back to 95% B in 0.2 min, followed by re-equilibration to give a total run time of 10 min.

The analytes were ionized in positive mode using a heated electrospray ionization (HESI) probe. The spray voltage, sheath gas, auxiliary gas, and sweep gas settings were optimized using the instrument’s optimizer. The final settings were 3.50 kV for spray voltage and 30, 15, and 0 arbitrary units for sheath, auxiliary, and sweep gas, respectively. Full MS and DIA-MS scans were acquired at various mass resolutions for optimization (see below). The scan range was set from *m/z* 75 to 550. A normalized collision energy (NCE) of 30 was applied for DIA-MS scans and lock mass correction by EASY-IC™ was enabled.

### Optimization of DIA-MS settings

For DIA-MS optimization, the scan parameters in Table 1 were evaluated. As these parameters affect the cycle time of the mass spectrometer and thus the number of measurements per peak, settings were selected to ensure that a minimum of 10 measurement points were obtained per chromatographic peak. The highest mass resolutions of MS1 and MS2 scans evaluated were 120.000 (resulting in an MS1 scan time of 288 ms) and 15.000 (42 ms/MS2 scan), respectively. To limit the isolation window widths of the DIA scans, multiplexing of the MS2 ions was employed when using high resolution MS1 scans. By multiplexing ions, i.e., collecting multiple MS2 ions prior to high-resolution mass determination, the number of required scans per cycle can be reduced, but at the cost of increased spectral complexity.

**Table 1:** Combinations of resolutions (MS1 and MS2), isolation window widths, and number of multiplexed scans tested for DIA optimization

Resolution		No. of MS2 windows	Isolation window width (Da)	Multiplex
**MS1**	**MS2**			
30.000	7.500	32	15	0
	15.000	16		
60.000	7.500	32	15	2
	15.000	16		
120.000	7.500	22	21	4
	15.000	11		

### Method validation

The linear working range, limit of detection, and limit of quantification were determined based on external standards. Since no analyte-free matrix was available, method validation experiments were performed with commercially available food samples, yeast extract, and soy sauce, respectively. Linear working ranges were determined from the calibration curves where the total peak area obtained from all selected DIA precursor and product ions for the analyte was plotted against the analyte concentration. LOD was determined based on the signal-to-noise (S/N) of 3, whereas LOQ was determined with an S/N ratio of 10.

To evaluate the precision, yeast extract and soy sauce were measured six times within a day (intra-day) and afterwards on two more days (inter-day) in triplicate. The instrument precision is reported as relative standard deviation (RSD%).

### Data and statistical analysis

Xcalibur v4.7.69.37 (ThermoFisher Scientific) was used for instrument control and data acquisition. DIA data was analyzed using Skyline 64-bit v24.1.0.414 (MacCoss Lab Software, Seattle, WA, USA) [22], with all integrated peaks being manually inspected to ensure correct peak detection and integration. A list containing retention times and the selected precursor and fragment ions of the peptide standards used was created using the experimentally determined MS/MS spectra from the measured standards. The list of precursor and product ions is shown in supplementary Table S2. The fragment ions selected for quantification were based on signal intensity and selectivity.

Compound Discoverer v3.3.2.31 (ThermoFisher Scientific) was used for untargeted data processing. An existing processing workflow “Food Research Unknown ID workflow with Online and Local Database searches” was used with the following modifications: For retention time alignment, the RT tolerance was set to 0.5 min; in the group compounds node, the peak rating threshold was set to 5 and the number of files to 3; the fill-gaps node was added to the workflow and 'Descriptive statistics', 'Differential analysis' were used as post-processing nodes. After application of the workflow, the coefficient of variance (%CV) was calculated (*n* = 3) and used for evaluation of the intermediate precision of the DIA optimization settings.

## Results and discussion

Among the various HILIC stationary phases, the zwitterionic phases are especially suited for the separation of highly polar, charged peptides. This is due to their unique combination of ionic and hydrophilic-partitioning retention mechanisms [23]. These properties are particularly important for sour, umami, and salt-tasting peptides, which commonly comprise one or more acidic amino acids (Asp/Glu) next to a small number of neutral amino acids in their sequence [6]. Accordingly, we selected a zwitterionic HILIC stationary phase for all subsequent experiments.

**Fig. 1:**
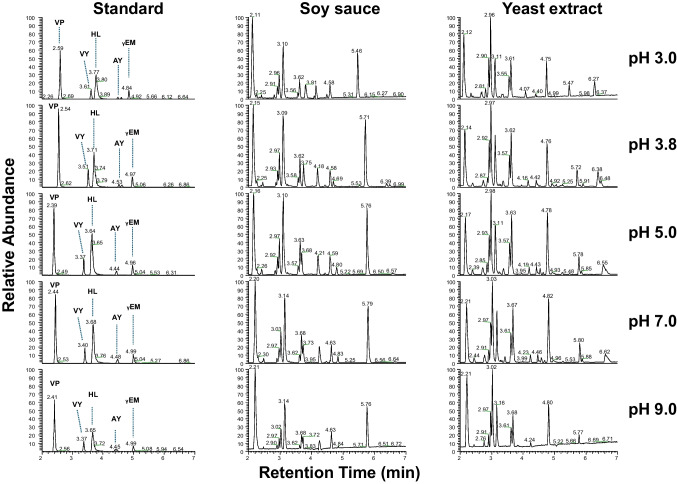
Base peak ion chromatograms (*m*/z 75 to 550) showing the effect of mobile phase buffer pH on the chromatographic behavior for a peptide standard mixture, soy sauce, and yeast extract sample**.** The chromatograms highlight separation characteristics across pH values ranging from pH 3.0 to 9.0

To optimize the separation, the pH, buffer concentration, column temperature, injection volume, and flow rate were evaluated. Changes in pH and buffer concentration had only a limited effect on the retention behavior of the peptides in the standard, as well as on the chromatographic profiles of soy sauce and yeast extract samples, as shown in Fig. 1. This was in line with expectations for the peptide VP in the standard, which contains neither charged nor polar residues. This compound showed unchanged retention across all pH values tested. For peptides containing acidic amino acids, a stronger pH-dependent retention was anticipated due to changes in the protonation state of Asp and Glu side chains. However, in short peptides, the overall net charge is often dominated by the protonated N-terminus and by basic side chains (e.g., Lys and Arg). As a result, changes in pH have only a limited influence on retention, as also observed by Di Palma (2011) [24].

Based on the buffering capacity of ammonium formate, a pH of 3.8 was considered optimal. Variations in buffer concentration had only a minimal impact on peptide retention (not shown). Higher buffer concentrations are preferred to improve buffering capacity and run–to–run reproducibility [25], but this likely results in a reduced MS signal intensity due to ion suppression caused by the buffer [26]. To balance buffering capacity and MS sensitivity, a buffer concentration of 10 mM was considered the best trade-off.

The optimal column temperature was found to be 50 °C. At lower temperatures, both peak shape and retention were negatively influenced. Especially the early-eluting nonpolar peptide VP showed much less retention at lower temperatures. Moreover, for the model peptide AY, peak splitting occurred below 50 °C, indicating the presence of multiple interaction modes with the stationary phase under these conditions.

Injection volumes above 2 µL caused overloading of early-eluting peaks due to a solvent mismatch with the initial gradient. To balance sensitivity and sample solubility, a 2 µL injection was selected, corresponding to about 1% of the column volume. Furthermore, a flow rate of 0.5 mL/min provided an effective separation with sharp peaks and a total analysis time of 10 min.

To summarize, the optimized HILIC conditions included an injection volume of 2.0 µL, a flow rate of 0.5 mL/min, and a column temperature of 50 °C. Solvent A consisted of 10 mM ammonium formate in ultrapure water. Solvent B was 10 mM ammonium formate in 95% ACN-ultrapure water. The pH of both solvents was set to pH 3.8 with formic acid prior to the addition of acetonitrile. A gradient separation was applied starting at 95% B, decreasing to 40% B in 7.5 min, back to 95% B in 0.2 min, followed by re-equilibration to give a total run time of 10 min. When operated at these optimized conditions, the zwitterionic HILIC column demonstrated excellent performance for the separation of short, polar and taste-active peptides. The strong retention of the HILIC phase is advantageous for separating salt- and umami-tasting peptides, which typically contain these acidic amino acids and are difficult to retain in reversed-phase LC.

### DIA optimization

Having established the chromatographic conditions for the rapid separation of polar taste-active peptides, we next focused on optimizing the Data-Independent Acquisition (DIA) mass spectrometry parameters. Optimizing DIA parameters such as scan cycle time, mass resolution settings, and isolation window width is crucial for maximizing MS/MS coverage and ensuring reproducible quantification with short total analysis times.

One key goal was to acquire 10 data points across each chromatographic peak to ensure robust quantification. In our experiments, the median LC peak width was ~ 7.6 s at baseline, so we targeted a total cycle time of ~ 760 ms per DIA cycle. Since orbitrap MS scans at the highest resolution require acquisition times exceeding this available time (supplementary Table S3), the DIA parameters need to be carefully balanced.

For DIA optimization, the MS1 and MS2 mass resolution, isolation window width, and multiplexing options were varied while maintaining a comparable MS cycle time. Under these conditions, increasing MS1 resolution increases the Orbitrap transient time, which reduces the number of MS2 scans that can be acquired per cycle. If this is not possible, wider and more multiplexed isolation windows must be used to maintain acceptable overall cycle times, yet this will adversely affect the precision of MS2 fragment-ion–based quantification. The settings explored are listed in Table 1.

**Fig. 2:**
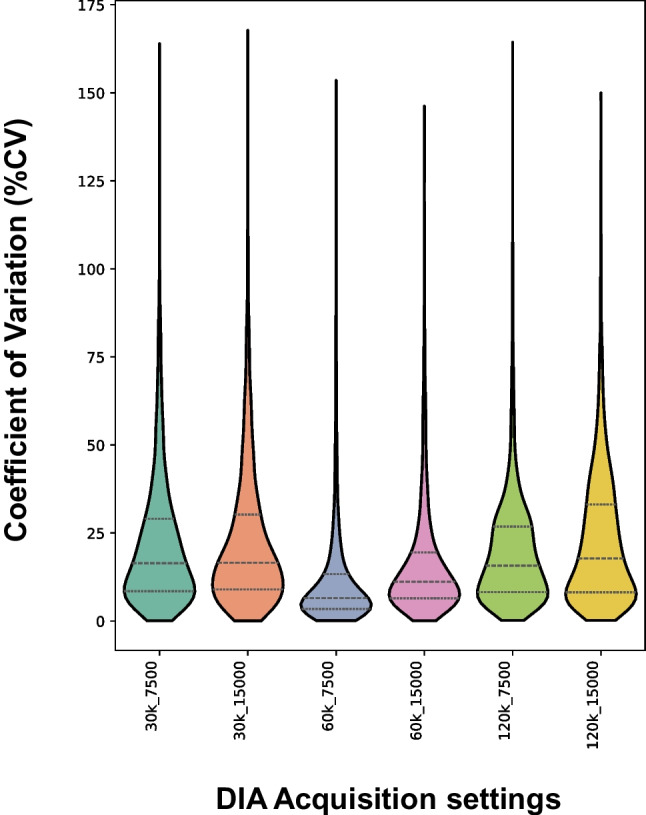
Violin plots visualizing the distribution of coefficient of variation (%CV) for 3910 detected features in a yeast extract under different DIA acquisition settings. Each label denotes the MS1 and MS2 resolution used. The width of each violin represents the frequency of observed %CVs. Lower and more compact distributions indicate higher quantitative precision

The impact of the settings on the quantitative performance was evaluated using a complex yeast digest sample. Across all tested conditions, 3910 features (*m/z* and retention time pairs) were consistently detected across all samples. The distributions of the %CV values are summarized as violin distribution plots in Fig. 2.

The choice of DIA scan parameters had a clear effect on precision. A narrow distribution of %CV values with a median close to zero indicates high run-to-run intra-day precision, whereas a broader distribution with a higher median %CV shows greater variability (i.e., lower precision). Notably, the resolution of the MS1 scan had a clear impact on the %CV; the poorest precision was observed with low-resolution (30.000) MS1 scans. Clearly, for these complex samples, enhanced mass separation at 60.000 or 120.000 mass resolution is beneficial because lower values allow isobaric molecules to significantly contribute to signal variability. Fast MS2 scans (7.500 resolution) proved advantageous in terms of quantitative variability at all MS1 resolutions. The optimal settings resulting in the lowest median %CV were obtained using an MS1 resolution of 60.000 in combination with rapid MS2 scans at 7.500. Measurements at a mass resolution of 120.000 required a higher degree of multiplexing of the MS2 ions to acquire data. The increased complexity of the spectra obtained here negatively influenced the %CV.

These results demonstrate that careful optimization of both MS1 and MS2 resolution parameters is critical for maximizing the precision of DIA-based peptide quantification.

**Fig. 3:**
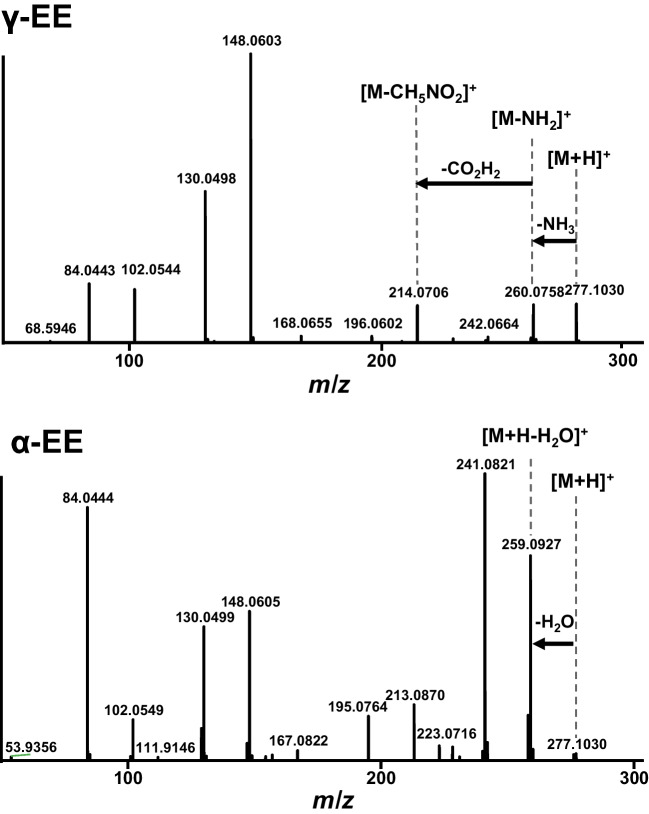
MS/MS spectra of the isomeric peptides γ-Glu-Glu (γ-EE) and α-Glu-Glu (α-EE). The specific fragmentation patterns annotated above the spectra provide a reliable basis for differentiating these isomers based on their unique fragments

While overall precision is a very important metric for method optimization, the analysis of specific peptide subclasses here introduces an additional challenge. The alpha-glutamyl (α-Glu-X) and gamma-glutamyl (γ-Glu-X) peptides are structural isomers yet differ in taste impact. α-Glu-X structures are linked to umami taste whereas γ-Glu-X structures are often associated with a kokumi perception [27]. Therefore, these two isomers need to be distinguished, preferably both by chromatographic separation and MS spectral differentiation. As shown in Fig. 3, the MS/MS spectra of γ-Glu-Glu (retention time 5.9 min) and α-Glu-Glu (retention time 6.1 min) reveal that several fragment ions derived from the C-terminal Glu are in common between the two isomers (e.g., *m/z* 148.06, 130.05, 102.05, and 84.04). However, differences still exist between the isomers. For α-Glu-X, a unique loss of water (–18 Da) is observed, while γ-Glu-X is characterized by the loss of 17 Da [M + H-NH_3_]^+^ and 63 Da [M-CH_5_NO_2_]^+^, which are absent in the α-Glu-X spectra. Jointly with the slight difference in HILIC retention, these distinct fragmentation patterns provide a reliable basis for distinguishing these isomers.

**Fig. 4:**
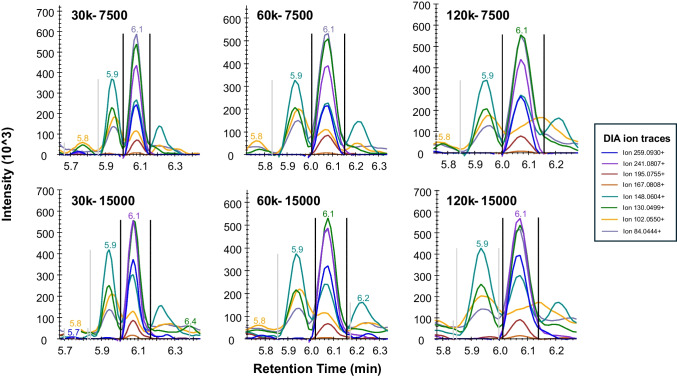
Extracted ion chromatograms of α-Glu-Glu product ions (retention time 6.1 min) acquired under various DIA settings in a yeast extract sample. The MS1 resolution varied between 30.000, 60.000, and 120.000, and the resolution of the MS2 scans varied between 7.500 and 15.000. The vertical black bars indicate the selected retention time window for peak integration. Details on applied isolation windows and multiplexing are shown in Table 1

To assess how the DIA settings influence the quantification and discrimination of isomeric peptides in a complex food matrix, we examined the performance of the DIA method settings on γ-Glu-Glu and α-Glu-Glu peptides in yeast extract. The DIA-MS product ion traces for α-Glu-Glu under different acquisition settings are shown in Fig. 4.

In DIA, quantification relies on the combined signal of all selected product ion traces. At a high MS1 resolution of 120.000, we observed that the intensity and shape of certain fragment ion signals (e.g., the yellow (*m/z* 102.055) and grey (*m/z* 84.044) traces) were altered and signals often failed to return to baseline compared to acquisition at lower MS1 resolutions. This effect is likely due to the use of wider DIA isolation windows required to maintain sufficient sampling points per peak at higher resolution, resulting in increased interference from co-isolated peptides and was also observed for γ-Glu-Glu (supplementary Fig. S1).

Given the identical precursor ions and closely related product ion *m/z* values of α-Glu–X and γ-Glu–X isomers, semi-quantitative analysis requires careful selection of product ions. Furthermore, the DIA precursor isolation window must be sufficiently narrow (≤ 16 Da) to minimize overlap from non-isomeric co-eluting species. For example, at wider isolation windows, the common neutral loss of water (18 Da) can lead to fragment ion overlap and interfere with quantification.

Although isomers may in some cases be distinguished by diagnostic fragments, under the applied conditions α- and γ-Glu–X isomers share the same major fragment ions, as demonstrated in Fig. 3. Differentiation therefore relies largely on the chromatographic separation, as achieved for α-EE and γ-EE in a yeast extract sample under the optimized HILIC conditions (Fig. 4). While during optimization of the DIA-MS settings, low MS1 resolution (30.000) did not directly affect the integration of α- and γ-Glu-Glu peptides, it significantly reduced overall precision, as shown in Fig. 2. In contrast, the optimized DIA method (MS1 at 60.000; MS2 at 7.500; ≤ 16 Da isolation window) provided both a good quantitative precision (low %CVs) and reliable differentiation of the isomeric peptides.

In summary, systematic optimization demonstrates that DIA acquisition settings have a major impact on the overall quantitative performance, particularly for the challenging analysis of isomeric peptides. In this workflow, chromatographic separation can provide differentiation of isomeric peptides, whereas DIA-MS parameters primarily influence robustness and reproducibility by minimizing non-isomeric interferences. Based on these findings, we recommend a moderate MS1 resolution of 60.000, fast MS2 scans, and a precursor isolation window of no more than 16 Da. This combination provides an optimal balance, maximizing precision while supporting reliable identification and semi-quantification of peptide isomers in complex food matrices.

### Method validation targeted quantification

**Fig. 5:**
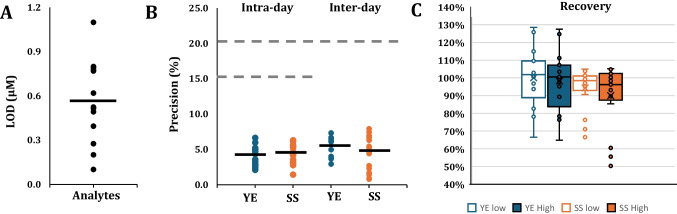
Method performance for semi-quantitative analysis of selected peptides in complex food matrices (*n* = 14) showing **A** limits of detection (LOD), **B** intra-day and inter-day precision (%RSD) of analyte quantification, and **C** recovery (%) after spiking in yeast extract (YE) and soy sauce (SS) samples at two concentration levels

Due to the lack of an analyte-free matrix, intra-day and inter-day precision were assessed using representative food matrices of a commercial yeast extract (YE) and soy sauce (SS). The corresponding results are summarized in Fig. 5. Limits of detection (LOD) and quantification (LOQ) were determined by stepwise dilution of a peptide standard mix, followed by HILIC-DIA-MS analysis. LOD and LOQ were defined as signal-to-noise ratios of 3 and 10, respectively, based on the selected MS2 fragment-ion traces. As shown in Fig. 5A, LOD values ranged from 0.10 to 1.10 µM and LOQ values from 0.30 to 3.50 µM, with median values of approximately 0.6 µM and 2.0 µM, respectively. These values are well below the taste threshold of relevant umami peptides in yeast [28].

All target analytes exhibited linearity with coefficients of determination *R*^2^ > 0.99, based on external calibration curves. The dynamic linear ranges varied among analytes spanning from 1.3–78.13 µM (γEE) to 3.0–372.57 µM (EE), as presented in supplementary Table S4. In Supplementary Fig. S3, chromatograms at LOQ level and representative calibration curves are shown.

The intra-day and inter-day precision assessments in YE and SS (Fig. 5B) showed excellent precision, with %RSD values ranging from 0.82 to 7.93%. All measurements fell well below established intra-day and inter-day performance thresholds of 15% and 20%, confirming the robustness of the approach for peptide quantification in food matrices. Retention times remained stable throughout the assessment; a chromatographic overlay of samples and standards is provided in Supplementary Fig. S2 to demonstrate this.

Recovery performance was evaluated by spiking YE and SS samples with target analytes at concentrations of 10–20 µM. Following sample preparation and HILIC-DIA-MS analysis, measured concentrations were compared to theoretical values. The overall average recovery across all analytes and matrices was 96%, demonstrating high extraction and quantification efficiency (Fig. 5C). However, some low recoveries were observed for γEF (50%), EL (60%), and γEI (56%) in the soy sauce matrix, while all other components were tightly distributed between 91% and 105%. In contrast, recoveries in yeast extract were more variable, ranging from 76% (γEI) up to 125% (EK). These results indicate that although the workflow is generally robust, recoveries are influenced by matrix-dependent interferences. The application of isotopically labeled internal standards is expected to further improve recovery accuracy, particularly for analytes showing matrix-specific suppression or enhancement effects.

### Method application on food samples

**Fig. 6:**
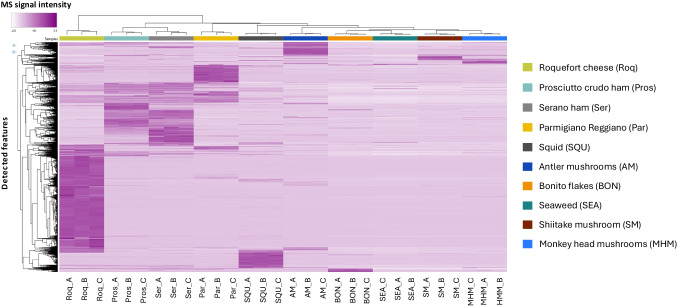
Hierarchical clustering analysis plot of untargeted DIA-MS features obtained from the analysis (*n* = 3) of food extracts by the developed HILIC-DIA-MS method. Samples are presented in the individual columns using colored indicators; vertically, the detected features are presented. The color scale indicates the measured MS intensity of each detected feature. The analysis illustrates overall similarity and differences in feature patterns between samples rather than identifying individual peptides responsible for these differences

Application of the HILIC-DIA-MS workflow to food products enabled both targeted data processing for semi-quantitative analysis of selected taste-relevant peptides (Table 2), as well as untargeted profiling of additional polar peptides and other molecular features within the same dataset (Fig. 6).

The cheese and ham samples analyzed yielded measurable concentrations of multiple taste-relevant peptides (Table 2). Cheese exhibited high levels of peptides formed through the extensive breakdown of caseins during ripening. Both α- and γ-glutamyl peptides were identified and quantified in these extracts. The presence of γ-glutamyl peptides, especially in Roquefort cheese (Roq), prosciutto ham (Pros), and Serrano ham (SH), is consistent with reported literature. Blue mold cheeses naturally contain intrinsic levels of γ-glutamyl peptides due to mold metabolism [29]. In Pros and SH, these peptides are likely generated through endo- and exopeptidases, such as dipeptidyl peptidases and gamma-glutamyl transferase during the curing process [30, 31]. Considering the dilution factors applied during the food extract preparation, the measured concentrations align well with literature values, confirming both the accuracy and robustness of the analytical workflow.

**Table 2:** Concentrations of selected taste-active peptides (µM) in analyzed food extracts, reported as mean ± standard deviation (*n* = 3)

Sample code	EE	EL	EV	DR	VF	EA	EK	γET	γEF	γEE	ED	DA	γEI	pEP
Roq	206 ± 10	140 ± 8	14 ± 1	2 ± 0	4 ± 0	-	322 ± 45	23 ± 1	10 ± 1	32 ± 1	29 ± 1	423 ± 37	26 ± 1	5 ± 0
Pros	61 ± 3	13 ± 0	3 ± 0	47 ± 2	5 ± 0	19 ± 1	69 ± 5	4 ± 0	-	15 ± 1	16 ± 2	40 ± 2	5 ± 0	1 ± 0
Ser	61 ± 4	4 ± 0	2 ± 0	55 ± 3		21 ± 1	61 ± 3	7 ± 1	1 ± 0	31 ± 2	15 ± 1	60 ± 3	9 ± 0	1 ± 0
AM	-	12 ± 1	2 ± 0	8 ± 0	6 ± 1	16 ± 1	14 ± 0	-	-	-	-	44 ± 0	-	-
SQU	26 ± 0	6 ± 0	-	1 ± 0	-	-	-	9 ± 1	-	7 ± 0	-	-	5 ± 0	-

- indicates below the limit of quantification

In the extracts obtained from dried ingredients (e.g., mushrooms, squid, seaweed, and bonito flakes), peptide levels were generally lower or not detectable, which is consistent with expectations given the mild extraction conditions applied. Among the 14 umami and kokumi peptide standards, several peptides were found in deer antler mushroom (AM) and in dried squid extracts (SQU). The liquid extract prepared from AM contained measurable amounts of the taste-enhancing peptides DA, EK, EA, VF, DR, EL, and EV. Yang [32] reported that deer antler mushroom had the highest umami intensity among eight edible fungi tested, likely due to the presence of these peptides. Similarly, the peptides in SQU (EE, γEE, γEI, γET, DR, and EL) are consistent with expectations, as the drying process facilitates their formation through complex enzymatic reactions associated with protein degradation [33]. The targeted data processing demonstrates that the concentrations of relevant peptides can be semi-quantified across diverse food types.

Importantly, the aqueous extraction approach was intentionally chosen to reflect common culinary practices, where dried seafood and mushrooms are infused to release flavor. In such cases, taste perception is likely driven not only by peptides but also by free amino acids and nucleotides, both known contributors to umami taste enhancement [34].

Although these additional compounds, along with non-targeted peptides, are not directly included in the presented targeted analysis (Table 2), they can be readily captured through the broader untargeted evaluation of the DIA datasets, as discussed below.

To extend the analytical coverage, the scope of the targeted data processing can be expanded by incorporating the fragmentation patterns of additional peptides into the predefined DIA analyte list (supplementary Table S2), enabling analysis of these features within previously acquired DIA datasets.

Next to targeted data processing using a predefined analyte list, the acquired DIA data can also be subjected to fully untargeted data interrogation. To this end, hierarchical clustering analysis of all detected features in the untargeted DIA data was performed, revealing distinct patterns characteristic of the various samples (Fig. 6). Closely related products, such as Serrano (Ser) and prosciutto ham (Pros), are clustered together, while Roquefort cheese (Roq) separates into its own branch, highlighting its distinct and complex composition [35]. Sample extracts of squid (SQU), bonito (BON), seaweed (SEA), and mushrooms (AM, SM, MHM) also form clearly defined groups, where SQU and AM both show highly characteristic molecular signatures. Several intense features (deep purple color) were uniquely associated with specific samples, suggesting potential markers for authentication studies or flavor-relevant investigations.

It should be noted that these samples were subjected to different preparation procedures (solid food homogenates versus aqueous extracts), which may contribute to some of the observed grouping. The results presented here are not intended as an exhaustive profiling of the selected food products, but rather to demonstrate the potential of the approach. They highlight the added value of applying an optimized HILIC-DIA-MS method. This strategy not only delivers strong, reproducible semi-quantification of known taste-relevant peptides but also enables untargeted profiling of the broader molecular landscape, providing comprehensive fingerprints that support product characterization, authenticity assessment, and flavor development. Together, these complementary strengths underscore the power of HILIC-DIA-MS in advancing food peptide research.

## Conclusion

An optimized HILIC-DIA-MS workflow was developed for the analysis of short polar peptides in complex food matrices. Optimization of chromatographic conditions and DIA acquisition parameters resulted in a method that combines reproducible semi-quantification of known taste-relevant peptides with broad untargeted coverage. Validation in representative food matrices confirmed adequate sensitivity, precision, and robustness. Application to diverse foods demonstrated accurate semi-quantification while simultaneously revealing distinct molecular fingerprints that differentiate between sample types. A key advantage of the developed DIA-based workflow is the ability to re-process untargeted DIA datasets post-acquisition for additional peptides and molecular features without the need for re-analysis. The combined strength of targeted and untargeted profiling highlights the potential of this workflow for food authentication, quality assessment, and flavor research. This work demonstrates that untargeted profiling of polar peptides in foods by HILIC-DIA-MS provides both accurate semi-quantification and comprehensive molecular characterization.

## Supplementary Information

Below is the link to the electronic supplementary material.

Supplementary file1 (DOCX 897 KB)

## Data Availability

Data will be made available on request.

## Declarations

### Generative AI and AI-assisted technologies in the writing process

During the preparation of this work, the author(s) used Microsoft Co-pilot (GPT-4) to improve the readability and language of the manuscript. After using this tool/service, the author(s) reviewed and edited the content as needed and take(s) full responsibility for the content of the publication.
