# Supplementary material for: Integrated targeted and untargeted analysis of polar peptides in foods using hydrophilic interaction liquid chromatography–data-independent acquisition–mass spectrometry
Source: Anal Bioanal Chem. 2026 Jan 28;418(10):3013–24. doi: 10.1007/s00216-026-06322-7 (PMC13144269; doi:10.1007/s00216-026-06322-7)
Supplement: Supplementary file 1 — Supplementary file1 (DOCX 897 KB) [file 216_2026_6322_MOESM1_ESM.docx]

**Supplementary material**

**Integrated targeted and untargeted analysis of polar peptides in foods using Hydrophilic Interaction Liquid Chromatography–Data-Independent Acquisition–Mass Spectrometry**

Boudewijn Hollebrands*^a,b^, Germaine Thong^a^, Hans-Gerd Janssen^a,b^

^a^Unilever Foods Innovation Centre-Hive, Wageningen, The Netherlands

^b^Wageningen University & Research, Laboratory of Organic Chemistry, Wageningen, The Netherlands

*Corresponding author:
Boudewijn Hollebrands

Unilever Foods Innovation Centre-Hive

Bronland 14

6708 WH Wageningen, the Netherlands

e-mail address: boudewijn.hollebrands@unilever.com

**Table of contents**

| Figure S1 | 3 |
| --- | --- |
| Figure S2 | 4 |
| Figure S3 | 5 |
| Table S1 | 6 |
| Table S2 | 7 |
| Table S3 | 8 |
| Table S4 | 9 |
| Protocol for HILIC Eluent Preparation | 11 |


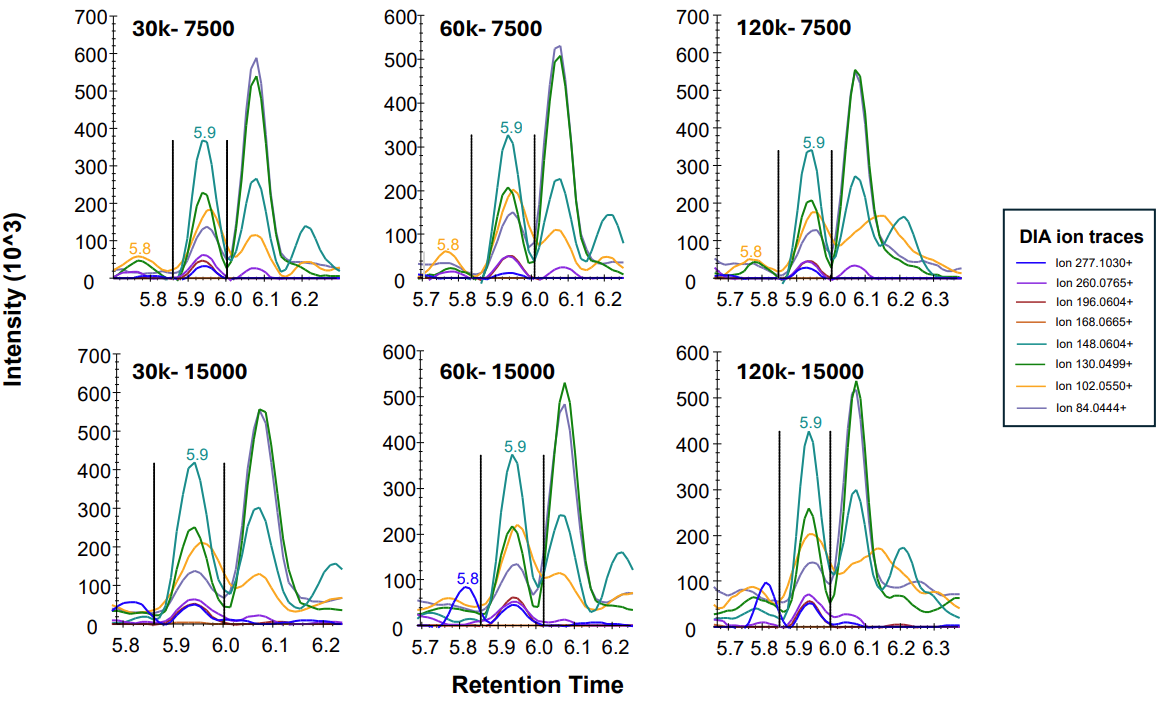


**Figure S1**. *Extracted ion chromatograms of* γ-*Glu-Glu product ions (retention time 5.9 minutes) acquired under various DIA settings in a yeast extract sample. The MS1 resolution varied between 30.000, 60.000 and 120.000 and the resolution of the MS2 scans varied between 7.500 and 15.000. The vertical black bars indicate the selected retention time window for peak integration. Details on applied isolation window width and multiplexing settings are shown in* ***Table 1****.*


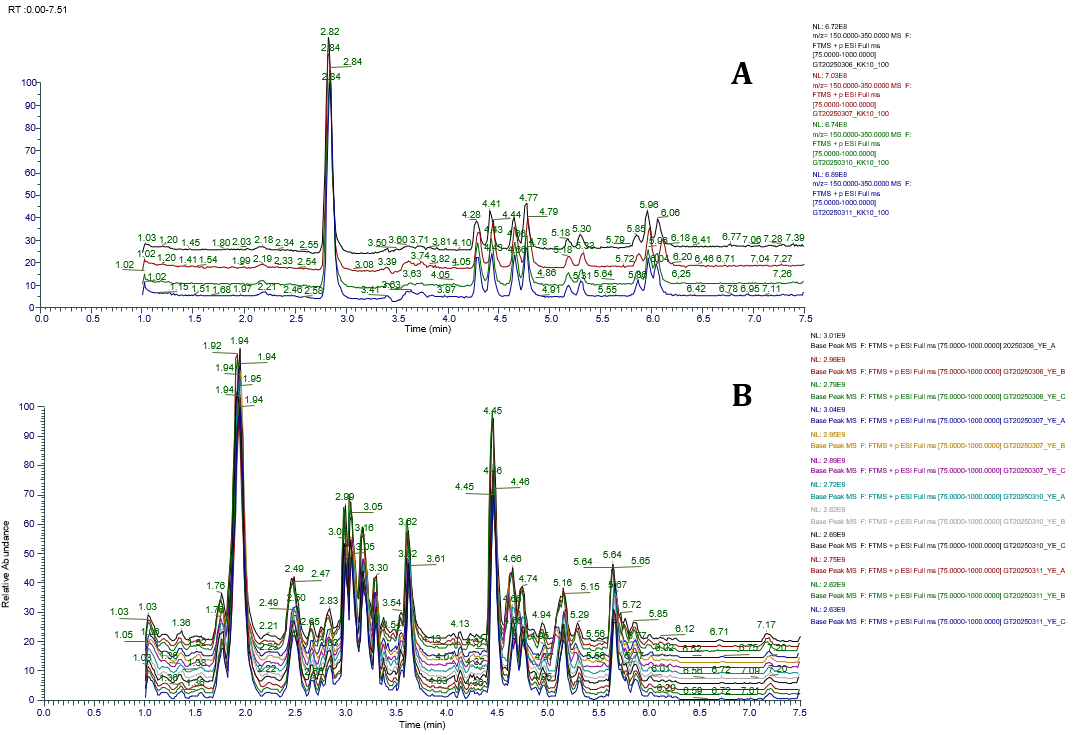


**Figure S2**. *Overlay of the base peak ion chromatograms of peptide standards (****A****) and yeast extract samples (****B****) acquired over 4-days during validation, demonstrating the retention time stability.*


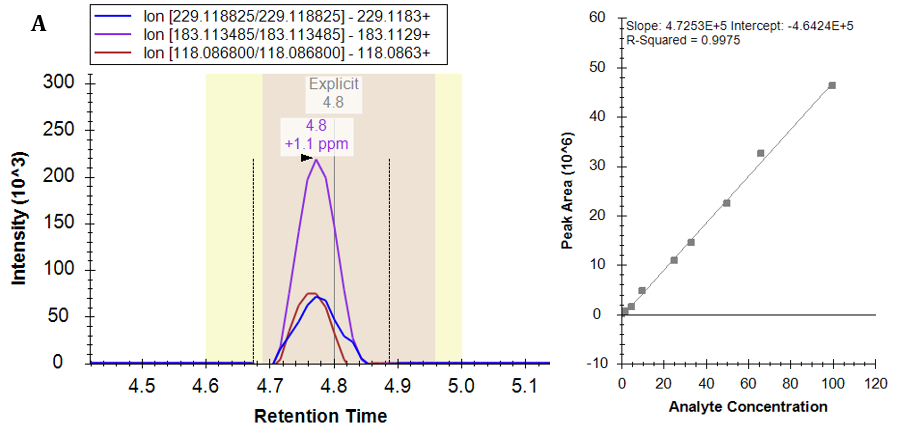


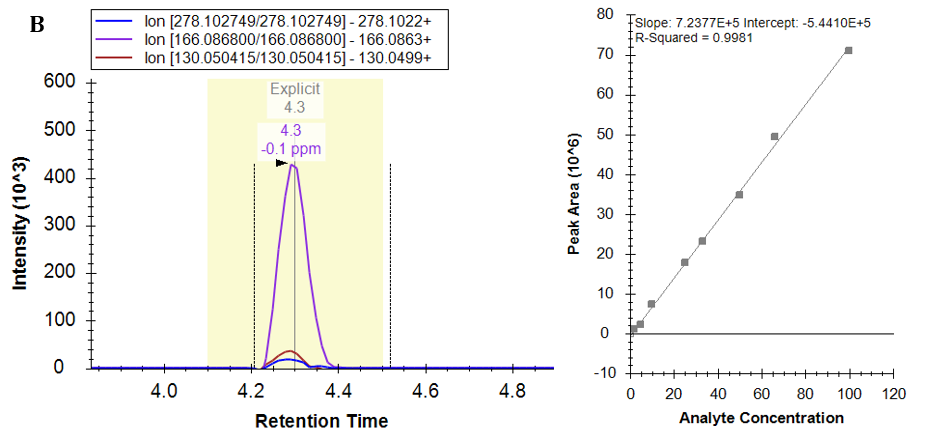

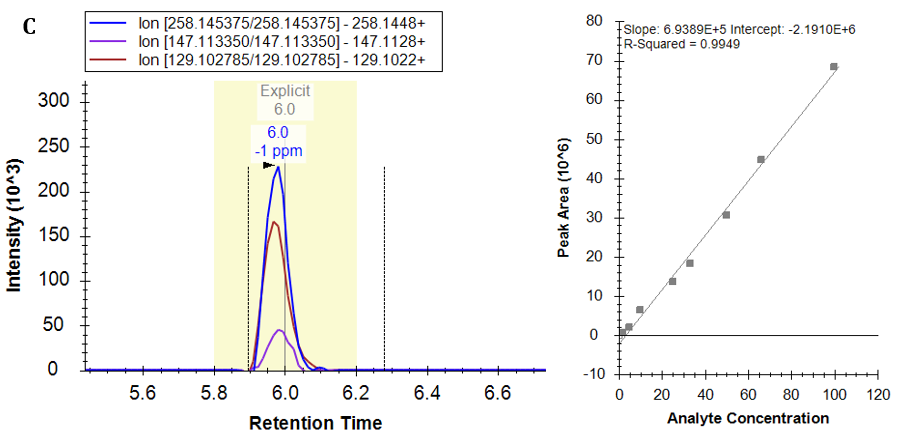


**Figure S3**. *Exemplary chromatograms of lowest measured concentration of the standards EV (****A****), γEF (****B****) and EK (****C****) and their respective calibration curves using the selective fragment ions listed in Table S2.*

**Table S1.** *Overview of commercial food samples analysed.*

| **Sample Code** | **Sample Name** | **Category** | **Type** | **Country** |
| --- | --- | --- | --- | --- |
| Roq | Roquefort | Cheese | Soft – 50+ | France |
| Par | Parmigiano Reggiano | Cheese | Hard – 24 months | Italy |
| Pros | Prosciutto Crudo | Dry-Cured Ham | Solid | Italy |
| Ser | Serrano Gran Reserva | Dry-Cured Ham | Solid | Spain |
| YE | Yeast Extract | Sauce & Seasoning | Powder | France |
| SS | Soy Sauce | Sauce & Seasoning | Liquid | China |
| SM | Shiitake Mushroom | Water Extract | Dried | China |
| MHM | Monkey Head Mushroom | Water Extract | Dried | China |
| AM | Deer Antler Mushroom | Water Extract | Dried | China |
| Bon | Bonito Flakes | Water Extract | Dried | France |
| Squ | Glassy dried Squid | Water Extract | Dried | Thailand |
| Sea | Dried Seaweed | Water Extract | Dried | South Korea |

**Table S2.** *List of precursor ions, production ions and retention times of 14 peptide standards.*

| **Analyte** | **RT** | **[M+H]^+^** | **Fragment Ions (*m/z*)** | | | | | | | | | | |
| --- | --- | --- | --- | --- | --- | --- | --- | --- | --- | --- | --- | --- | --- |
|  | **(min)** |  |  |  |  |  |  |  |  |  |  |  |  |
| **EE** | 6.05 | 277.1030 | 259.09 | 241.08 | 148.06 | 130.05 | 102.06 | 84.04 |  |  |  |  |  |
| **EL** | 4.40 | 261.1445 | 243.13 | 225.12 | 197.13 | 132.10 | 102.05 | 86.10 | 84.04 |  |  |  |  |
| **EV** | 4.80 | 247.1445 | 229.12 | 211.11 | 183.11 | 155.12 | 118.09 | 102.05 | 84.04 | 72.08 |  |  |  |
| **DR** | 5.90 | 290.1459 | 273.12 | 175.12 | 158.09 | 158.06 | 116.07 | 112.09 | 70.07 | 60.06 |  |  |  |
| **VF** | 2.80 | 265.1547 | 166.09 | 120.08 | 72.08 | 55.05 |  |  |  |  |  |  |  |
| **EA** | 5.30 | 219.0975 | 201.09 | 155.08 | 90.06 |  |  |  |  |  |  |  |  |
| **EK** | 6.00 | 276.1554 | 258.14 | 240.13 | 222.12 | 195.11 | 157.10 | 147.11 | 130.09 | 129.10 | 102.05 | 84.08 |  |
| **ED** | 6.10 | 263.0874 | 245.08 | 227.07 | 199.07 | 181.06 | 134.04 | 116.03 | 102.05 | 88.04 | 84.04 |  |  |
| **pEP** | 3.65 | 227.1026 | 209.09 | 181.10 | 116.07 | 84.04 | 70.07 |  |  |  |  |  |  |
| **gET** | 5.20 | 249.1081 | 231.10 | 186.07 | 130.05 | 120.07 | 102.07 | 84.04 | 74.06 | 56.05 |  |  |  |
| **gEF** | 4.30 | 295.1288 | 278.10 | 232.10 | 186.09 | 166.09 | 149.06 | 130.05 | 130.05 | 120.08 | 84.04 |  |  |
| **gEE** | 5.95 | 277.1030 | 260.08 | 214.07 | 196.06 | 148.06 | 130.05 | 102.05 | 84.04 |  |  |  |  |
| **gEI** | 4.35 | 261.1445 | 244.12 | 198.11 | 130.05 | 86.10 |  |  |  |  |  |  |  |
| **DA** | 5.30 | 205.0819 | 187.07 | 170.04 | 159.08 | 145.06 | 141.07 | 124.04 | 113.07 | 99.06 | 90.05 | 88.04 | 70.03 |

*Selective fragment ions are indicated with an underscore*

**Table S3**. *Experimental scan times for different MS1 and MS2 scans performed at various mass resolutions.*

| **MS1 Resolution** | **Time (ms)** | **MS2 Resolution** | **Time (ms)** |
| --- | --- | --- | --- |
| 30.000 | 72 | 7.500 | 21 |
| 60.000 | 144 | 15.000 | 42 |
| 120.000 | 288 | 30.000 | 84 |

**Table S4.** *Standard* *purity, Linear Working Range (LWR) calibration curves and coefficient of determination (R^2^) of 14 umami and kokumi peptide standards. Limit of detection (LOD) and limit of quantification (LOQ). Precision after spiking in Soy Sauce (SS) and Yeast Extract (YE).*

| **No** | **Analyte** | **Purity (%)** | **LWR**  **(µM)** | **R^2^** | **LOD**  **(µM)** | **LOQ**  **(µM)** | **Precision (%) SS** | **Precision (%) YE** |
| --- | --- | --- | --- | --- | --- | --- | --- | --- |
| 1 | EE | 98.0 | 3.0 – 373 | 0.999 | 0.92 | 3.07 | 103 | 108 |
| 2 | EL | 99.6 | 2.0 – 199 | 0.993 | 0.62 | 2.07 | 68 | 94 |
| 3 | EV | 99.7 | 1.7 – 208 | 0.995 | 0.52 | 1.72 | 93 | 98 |
| 4 | DR | 98.8 | 0.9 – 73.4 | 0.993 | 0.28 | 0.92 | 97 | 113 |
| 5 | VF | 99.7 | 0.5 – 195 | 0.999 | 0.14 | 0.45 | 98 | 101 |
| 6 | EA | 99.8 | 2.4 – 81.0 | 0.998 | 0.72 | 2.41 | 114 | 104 |
| 7 | EK | 99.8 | 2.6 – 161 | 0.990 | 0.79 | 2.64 | 104 | 108 |
| 8 | ED | 96.0 | 2.6 – 82 | 0.995 | 0.77 | 2.56 | 94 | 100 |
| 9 | pEP | 99.8 | 0.3 – 95 | 1.000 | 0.10 | 0.34 | 97 | 66 |
| 10 | gET | 98.4 | 1.6 – 195 | 0.992 | 0.48 | 1.61 | 95 | 93 |
| 11 | gEF | 99.8 | 0.7 – 198 | 0.995 | 0.20 | 0.67 | 58 | 81 |
| 12 | gEE | 99.2 | 1.3 – 78.1 | 0.998 | 0.39 | 1.32 | 98 | 93 |
| 13 | gEI | 97.8 | 1.5 – 174 | 0.995 | 0.46 | 1.53 | 63 | 77 |
| 14 | DA | 99.5 | 2.5 – 105 | 0.997 | 0.76 | 2.52 | 98 | 105 |

**Protocol for HILIC Eluent Preparation**

**Preparation of Eluent A - 10 mM Ammonium Formate Solution (pH 3.80)**

**1)** Accurately weigh 630 mg ammonium formate using a precision balance and weighing paper. Transfer the weighed ammonium formate into a cleaned 1-liter eluent flask.

**2)** Add ~500 mL of Millipore water to the flask. Swirl the flask by hand until the added ammonium formate is dissolved.

**3)** Continue adding Millipore water until the total volume reaches 1.000 L.

**4)** Stir the contents of the flask thoroughly to ensure the solution is homogeneous.

**5)** Adjust the solution to pH 3.80 by gradually adding formic acid (≥99% LC–MS grade). After each addition, mix thoroughly and measure the pH using a calibrated pH meter, allowing the reading to stabilize.

**6)** Once the pH has stabilized at 3.80, the eluent is ready for use. Label the flask accordingly and store it under appropriate conditions if not used immediately.

**Preparation of Eluent B - 10 mM Ammonium Formate Solution in 95% acetonitrile**

**1)** Transfer 50 mL of Eluent A, the 10 mM Ammonium Formate Solution (pH 3.80), into a clean 1-liter eluent flask using a graduated cylinder.

**2)** Accurately weigh 598.5 mg of ammonium formate using a precision balance and weighing paper. Add the ammonium formate to the eluent flask and swirl or stir until completely dissolved.

**3)** Adjust the solution to pH 3.80 by gradually adding formic acid (≥99% LC–MS grade). After each addition, mix thoroughly and measure the pH using a calibrated pH meter, allowing the reading to stabilize.

**4)** Transfer the contents of the flask into a 1 L graduated cylinder.

**5)** Add approximately 200 mL of acetonitrile (LC–MS grade) and mix gently to homogenize.

*Note: Temporary cloudiness may occur due to reduced salt solubility in high acetonitrile content. Add acetonitrile gradually with mixing. If cloudiness persists, allow the solution to equilibrate and gently warm (hand-warm) until the solution becomes clear.*

**6)** Continue adding acetonitrile in small portions while mixing until the total volume reaches 1.000 L.

**7)** Transfer the solution back into the eluent flask. Label the flask with composition and preparation date and store under appropriate conditions until use.
